# Trajectories of patients with severe mental illness in two-year contact with Flexible Assertive Community Treatment teams using Routine Outcome Monitoring data: An observational study

**DOI:** 10.1371/journal.pone.0207680

**Published:** 2019-01-09

**Authors:** Hans Kortrijk, Barbara Schaefer, Jaap van Weeghel, Cornelis L. Mulder, Astrid Kamperman

**Affiliations:** 1 Parnassia Group, Bavo-Europoort Mental Healthcare Organization, TA Rotterdam, The Netherlands; 2 GGZ Delfland Mental Healthcare Organization, PL, Spijkenisse, The Netherlands; 3 Parnassia Group, Dijk en Duin Mental Health Centre, AH Castricum, The Netherlands; 4 Tilburg University, Department of Social and Behavioural Sciences, Tranzo Scientific Centre for Care and Welfare, LE Tilburg, The Netherlands; 5 Phrenos Centre of Expertise, BE Utrecht, The Netherlands; 6 ESPRI Epidemiological and Social Psychiatric Research Institute, Department of Psychiatry, Erasmus Medical Center, CA Rotterdam, The Netherlands; Simon Fraser University, UNITED STATES

## Abstract

**Objective:**

Using outcome data collected routinely over a continuous two-year treatment period, we wished to distinguish homogeneous subgroups of patients with a severe mental illness whose psychosocial problems followed a similar pattern over time. By identifying the effectiveness of health services for different patient groups, this approach allowed us to identify patients at risk of deterioration and those recovering from their symptoms.

**Methods:**

In total we included 2,660 patients who were in two-year continuous contact with a Flexible Assertive Community Treatment team (FACT). We collected outcome data on psychosocial functioning, needs for care and quality of life. We performed a latent class growth analysis (LCGA).

**Results:**

The LCGA identified six homogenous patient subgroups using trajectories of HoNOS scores. On the basis of the patterns of patients’ psychosocial problems over time, we labelled these as follows: 1) stable at a low problem-severity level (N = 709; 27%); 2) stable at a low medium problem-severity level (N = 1,208; 45%); 3) stable at a high medium problem-severity level (N = 528; 20%); 4) stable at a high problem-severity level (N = 116; 4%); 5) amelioration of problems (N = 42; 2%); and 6) deterioration of problems (N = 57; 2%). Patients with stable and a high severity of psychosocial problems had more practical and somatic unmet needs than those in other subgroups, and also had the fewest decrease in the number of unmet needs.

**Discussion:**

After linking patient subgroups with clinical features such as the need for care, we found that, over two years, most patients remained relatively stable in terms of psychosocial functioning, but that their unmet needs decreased over time. However, in terms of needs for treatment during two years of contact with a FACT team, patients in the subgroup with a stable and high problem-severity level tended to derive little or no benefit.

## Introduction

The routine measurement of health outcomes has gained momentum over the past decade. Now, in 2018, Routine Outcome Monitoring (ROM) is a widely recognized standard around the world [1–2]. Using outcome data to determine effectiveness of healthcare services through benchmarking is nonetheless controversial [3–11]. Mostly because benchmarking has not yet fulfilled its promises, especially for patients with a severe mental illness (SMI) it has delivered hardly any clinically relevant improvements in treatment.

An important issue in this discussion is that the current analytic approaches to dealing with ROM data do not adequately describe the whole range of patient outcomes over time, i.e. the heterogeneity within patient trajectories. Most studies present patient outcomes in group summaries, using mean total scores or change scores over time. While this approach is appealing, as it is both easy to calculate and to understand, experience with SMI patients has shown that the plotted average outcomes usually result in a ‘flat line’ over time [7]. Below this stable average outcome, some patients improve, while others deteriorate or suffer chronically from severe psychiatric problems. In this patient group, this ‘group as a whole’ approach thus obscures the variations in outcomes, making it difficult to extract clinically relevant results from which we can learn and improve treatment.

Another common approach is to categorize and summarize individual outcomes (e.g. remission, recovery, reliable and clinically significant change [12–15]). This approach is more popular among clinicians, as it focuses on the individual patient and uses a recognizable and therapeutically relevant outcome. Usually, however, it uses only one baseline- and one follow-up measurement, thereby discarding all other potentially relevant measurements made on other occasions. This is problematic for SMI patients, who are characterised by long-lasting psychiatric problems. In SMI patients, these outcomes are also rather conservative, that is only a small proportion of patients gains a reliable improvement (calculated by using the reliable change index for example), and a low sensitivity to change is an important limitation for evaluating group outcomes [7].

In this paper we therefore focused on extracting clinically relevant results from routinely collected outcome data. Using latent class growth analysis (LCGA), we aimed to distinguish homogeneous subgroups of SMI patients whose psychosocial problems had followed similar patterns over a two-year treatment period. In line with previous studies, we expect to find a large subgroup of patients whose psychosocial problems had remained stable. But we also expected to find smaller subgroups of patients whose problems decreased or increased over the course of treatment.

To identify the effectiveness of health services for different patient groups, we also aimed to describe these patient subgroups in terms of socio-demographic and clinical characteristics, such as treatment needs and changes in these needs. Additionally, we hoped that this would help us identify patients who were recovering from their symptoms or were at risk of deterioration, this helps patients and clinicians to develop a more individually tailored treatment plan.

## Materials and methods

### Study setting and data collection

This study involved patients from two mental health institutes in the Netherlands, Bavo Europoort and Dijk en Duin. We included patients from 29 Flexible Assertive Community Treatment (FACT; [16]) teams that covered three geographical areas: the Greater Rotterdam Area (Bavo Europoort: 20 FACT teams); Midden-Kennemerland and Zaanstreek-Waterland (Dijk en Duin; 9 FACT teams).

FACT teams deliver rehabilitation-oriented clinical case management for patients with a SMI. Depending on the patient’s needs, each FACT team provides individual case management or a more assertive outreach with a shared caseload [16]. If a patient is unstable, at risk of relapse or readmission, or in the event of an admission to a psychiatric hospital, a court order, or at intake the FACT team places this patient on an electronic board and discusses these patients daily and provides intensive assertive outreach care on the basis of a shared caseload where necessary. For more stable patients, the teams use individual case management to provide coordinated multidisciplinary treatment and care [16].There are two criteria for treatment by a FACT team: a) age 18 or older, and b) having an SMI, the latter being characterized by 1) a history of psychiatric illness or treatment for two years or more, and 2) functional disabilities. The term SMI usually applies to clients with a psychotic, severely anxious mood or personality disorder, with or without a co-morbid substance-use disorder [17].

The data for this study were collected in the context of a ROM procedure. ROM assessments were performed by mental-health professionals (such as nurses, psychologists and social workers), and were planned annually before treatment-plan evaluation. These assessments were used in clinical practice when discussing treatment progress with the patient.

The study did not require extra assessments of patients, and ROM data-collection was approved by Central Committee on Research Involving Human Subjects (https://www.sbggz.nl/Nieuws/Nieuws-detail?ContentItem=e95a8797-583e-4f10-9680-89e9b283242b). Data for this study were collected during the period from January 2011 to July 2016, and were used confidentially. All data were fully anonymized prior to access by the authors.

From the ROM dataset (N = 6,897 patients, with a total of 20,925 assessments), we selected patients that were treated in a FACT team for a continuous period of two years, operationalized as a series of at least three consecutive ROM assessments one year apart over a two-year treatment period. Consecutive assessments needed to be more than six months but less than 18 months apart. From the total of 6,897 patients, we included the ROM-assessments of 2,660 patients (39%). We included three assessments per patient. The mean interval between the first and second assessments was 11.9 months (SD = 3.0 months); between the second and third assessments it was 11.7 months (SD = 2.4 months). To estimate the potential impact of our selection process, selection analyses were conducted.

We compared the demographic and clinical characteristics (sex, age, diagnosis, and severity of psychosocial problems at first ROM assessment) of the selected patients (N = 2,660) and the non-selected patients (N = 4,237). This showed that a larger proportion of the unselected patients were female (45.4% versus 40.2%; p = < .001). Unselected patients were approximately one year younger than selected patients (mean 41.9 versus 43.2 year, p<0.001), fewer had been diagnosed with a psychotic disorder (55.0% versus 65.0%, p<0.001); and more had been diagnosed with an anxiety disorder(20.0% versus 17.2%, p = 0.003) and a mood disorder (27.2% versus 22.7%, p<0.001). Reported psychosocial problems were more severe in the unselected patients (HONOS mean score 13.9 (SD = 6.9) than in the selected patients (HONOS mean score 12.7 (SD = 6.3) (p<0.001). There were no differences regarding the presence of co-occurring substance abuse, or personality disorders. Unselected patients had had fewer ROM-assessments than selected patients (M = 2.2 (SD = 1.3) versus M = 4.4 (SD = 1.2), p<0.001). Finally, we used multivariate logistic regression analysis to calculate whether the selection of patients had had any overall differences in terms of demographic and clinical characteristics. The resulting model fitted the data and suggested the presence of a potential bias (χ^2^(8) 142.14; p < .001).

### Measures

#### Psychosocial problems

Psychosocial problems were assessed using the Health of the Nation Outcome Scales (HoNOS). Developed for routine use in measuring a range of mental health outcomes [18], the HoNOS consists of 12 clinician-rated items, each using a five-point scale (0 = “no problem” to 4 = “severe/very severe problem”), and thus yielding a total score ranging from 0 to 48. The psychometric properties of the English and Dutch HoNOS versions have been found acceptable [18–20]. To indicate the severity of the psychosocial problems, we calculated a HoNOS total score (sum score of items 1–12).

#### Need for treatment

The Camberwell Assessment of Needs Short Appraisal Schedule (CANSAS)–a modified version of the Camberwell Assessment of Need (CAN) [21]–consists of 22 items measuring health and social needs across several relevant domains. The possible ratings per item are no problem/no need; met need (no/moderate problem because of help given); or unmet need (current serious problem regardless of any help received). The reliability of the CANSAS is acceptable [22]. The clinician’s and patient’s perspectives can both be assessed. For this study we used a 27-item patient-rated version, which includes the original 22 items and a Dutch addendum consisting of five extra items: (1) paid job, (2) side- effects of medication, (3) recovery, (4) legal problems and (5) sleep problems. The addendum was rated in the same manner as the original items. The total number of unmet needs (range 0–27) and the individual items are used.

#### Quality of life

To measure subjective quality of life (QoL), we used a quality-of-life scale, the Cumulative Needs for Care Monitor (CNCM; assessed in Dutch) [23], which is based on the Lancashire Quality of Life Profile [24] and is very similar to the Manchester Short Assessment of Quality of Life scale (MANSA) [25]. It also has strong correlations with the Lancashire Quality of Life Profile [23], and consists of seven items [26] that are rated on a 7-point scale (1 = “Couldn’t be worse” to 7 = “Couldn’t be better”). We calculated a QoL total score (range 7–49).

#### Socio-demographic and clinical characteristics

We collected data on gender, age and diagnosis (according to the Diagnostic and Statistical Manual of Mental Disorders-IV (DSM IV). DSM-IV diagnoses were made by a psychiatrist or psychologist from the FACT team during the intake interview and where necessary, diagnoses were adjusted and/or added during the treatment process.

### Statistical analysis

To perform a LCGA, we used consecutive HONOS scores over a two-year period. LCGA is a subtype of growth-mixture modelling. The method is person centred (rather than variable centred) and can be used to detect homogeneous subtypes (or trajectories) of people in a longitudinal multivariable dataset [27–30]. The aim of this approach, which focuses on the relationships among individuals, is to classify individuals into distinct subgroup trajectories on the basis of individual response patterns, such that individuals within a group are more similar than individuals between groups. The optimal number of subgroups is determined by statistical-fit measures, and also by the conceptual and clinical justification of the model [31–32].

In this study, LCGA was used to obtain homogenous subgroups of SMI patients with distinct linear trajectories of psychosocial problems over a two-year treatment period. Analyses were conducted using HONOS total scores of 3 consecutive assessments per patient, which were used as inputs for LCGA. Since HONOS total scores were non-normally distributed, using robust maximum likelihood estimation (MLR) was used to correct for the bias caused by departure from normality in the standard errors [33]. The number of extracted subgroups of patients ranged between 2 and 8. To determine the optimal number of latent subgroups and the overall statistical fit of the model, we used several goodness-of-fit indices including Akaike’s information criterion (AIC)); (adjusted) Bayesian information criterion (BIC); entropy; the Lo-Mendell-Rubin likelihood ratio test (LMR-LRT); and bootstrap likelihood ratio test (bootstrapped LRT) [34–38]. We also took account of parsimony, theoretical justification and clinical interpretation of the distinct subgroups. We used Mplus v7.4 [39] to conduct the analyses.

Patients were allocated to a specific subgroup on the basis of their highest posterior subgroup probability, and the resulting subgroups were labelled on the basis of the patients’ psychosocial problem-severity level (HoNOS scores). Finally, to formally test potential associations between the identified subgroups and the demographic and clinical characteristics, we used Chi2-tests for categorical variables; and ANOVA’s and Kruskal-Wallis with post-hoc pairwise comparisons for continuous variables. Pearson (QoL) and Spearman rank (CANSAS) correlation tests were used to calculate correlations between HONOS scores, QoL scores and CANSAS scores at the different assessments. Differences in the number of needs between assessments 1 and 3 were analysed using Wilcoxon signed rank test for paired samples, and using the McNemar test for the presence of unique needs. Analyses were conducted using SPSS v21.0.

Due to our data-selection procedure, we had no missing HONOS data. Normality of the data was inspected visually using histograms and Q-Q plots, and statistically using Kolmogorov-Smirnov tests. With regard to the included patients’ demographic and clinical characteristics, 8.9% of data was missing. When testing the potential associations between the subgroups and demographic and clinical characteristics, we excluded cases with missing data from analysis.

## Results

### Patient characteristics

A majority of the 2,660 SMI patients who were selected for this study were male (N = 1,589; 59.7%). Patients’ mean age was 43.2 years (SD 10.8 years; min 18.3 years–max 85.0 years). Most had been diagnosed with a psychotic disorder (N = 1,729; 65%). Six hundred and thirty-three (24%) were diagnosed with a co-occurring substance-use-related disorder, and 604 (23%) with a mood disorder. Four hundred and fifty (17%) were diagnosed with an anxiety disorder and 417 (16%) with a personality disorder. Two hundred and ninety-nine (11%) had a deferred diagnosis on axis II (DSM IV).

### Homogeneous subgroups of SMI patients using latent class growth analysis

The results of the LCGA are reported in Table 1. AIC, BIC, and adjusted BIC decreased as the number of subgroups increased; bootstrapped LRT was significant for all models. Entropy reached its peak at six subgroups of patients with homogeneous linear trajectories, and the LMR-LRT suggested that eight and more subgroups of patients did not increase the fit of the model. A seven-subgroup model included a subgroup containing less than 1% of patients. Taking account of these goodness-of-fit measures, the parsimonious principle, the theoretical justification and clinical interpretation, we concluded that the data was best described by a model consisting of six homogeneous subgroups of patients based on their HoNOS-trajectory (AIC = 48,787; BIC = 48,905; adjusted BIC = 48,841; Entropy = 76%).

**Table 1:** Latent class growth analysis procedure and model fit.

Number of latent subgroups	2	3	4	5	6	7	8	9
AIC*	49727	49137	48957	48838	48787	48745	48722	48712
BIC*	49774	49202	49040	48938	48905	48880	48875	48883
Adjusted BIC*	49749	49167	48995	48885	48841	48807	48793	48790
Entropy	74%	74%	71%	75%	76%	75%	68%	69%
Lo, Mendell, Rubin LRT*	2 vs. 1 1782 P<0.00001	3 vs. 2 572 P<0.00001	4 vs. 3 178 P = 0.0010	5 vs. 4 120 P<0.00001	6 vs. 5 55 P = 0.0118	7 vs. 6 46 P = 0.0169	8 vs. 7 28 P = 0.3039	9 vs. 8 33 P = 0.3255
Bootstrapped LRT*	P<0.00001	P<0.00001	P<0.00001	P<0.00001	P<0.00001	P<0.00001	P<0.00001	P<0.00001
N for each class	1:N = 1792 (67%) 2:N = 868 (33%)	1:N = 1105(42%) 2:N = 1292 (48%) 3:N = 263 (10%)	1:N = 1234 (46%) 2:N = 110 (4%) 3:N = 562 (21%) 4:N = 754 (28%)	1:N = 1247 (47%) 2:N = 52 (2%) 3:N = 108 (4%) 4:N = 739 (28%) 5:N = 514 (19%)	1: N = 709 (27%) 2: N = 1208 (45%) 3: N = 116 (4%) 4: N = 42 (2%) 5: N = 528 (20%) 6: N = 57 (2%)	1: N = 667 (25%) 2: N = 526 (20%) 3: N = 39 (1%) 4: N = 47 (2%) 5: N = 145 (5%) 6: N = 73 (3%) 7: N = 1163 (44%)	1: N = 570 (21%) 2: N = 426 (16%) 3: N = 76 (3%) 4: N = 688 (26%) 5: N = 38 (1%) 6: N = 145 (5%) 7: N = 49 (2%) 8: N = 668 (25%)	1: N = 553 (21%) 2: N = 583 (22%) 3: N = 40 (25%) 4: N = 661 (25%) 5: N = 47 (2%) 6: N = 14 (1%) 7: N = 89 (3%) 8: N = 514 (19%) 9: N = 159 (6%)

* Performance measures: AIC: Akaike information criterion; BIC: Bayes information criterion; LRT: Likelihood Ratio Test

**Fig 1:**
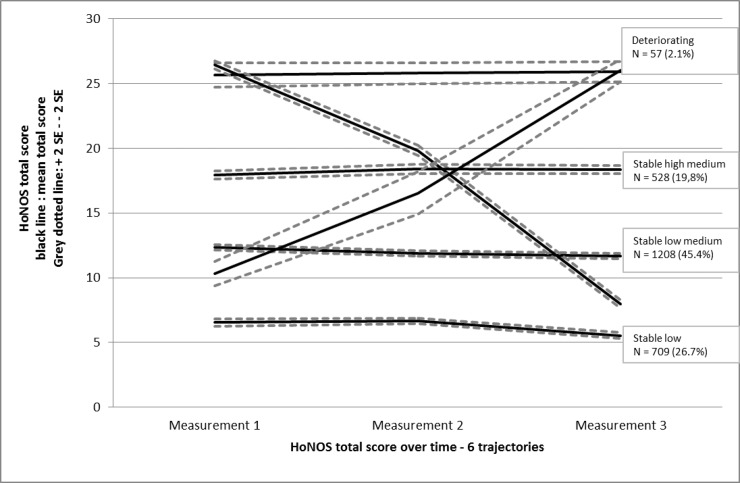
HoNOS total scores of six subgroups consisting of homogenous groups of SMI patients in contact with a FACT team over a two-year period.

Fig 1 shows the HoNOS scores for the patients in the six linear subgroup trajectories over the two years of treatment. On the basis of the patients’ severity of psychosocial problems over time, we labelled these six subgroup trajectories as follows: stable at a low problem-severity level (N = 709; 27%); stable at a low medium problem-severity level (N = 1208; 45%); stable at a high medium problem-severity level (N = 528; 20%); stable at a high problem-severity level (N = 116; 4%); amelioration of problems (N = 42; 2%); deterioration of problems (N = 57; 2%).

Table 2 shows that the subgroup of patients with a stable low HoNOS trajectory had the highest proportion of patients diagnosed with a psychotic disorder and also the lowest number of diagnoses per patient. On the other hand, patients in the subgroup with a stable high HoNOS trajectory and the subgroup of patients with a deteriorating HoNOS trajectory had a lower proportion of patients diagnosed with a psychotic disorder and a higher proportion of patients diagnosed with a substance-use disorder, personality disorder and anxiety disorder. Similarly, more patients with a stable high severity of problems had multiple diagnoses than other patients. Between the HoNOS subgroups there were no differences between patients in terms of gender or age.

**Table 2:** Clinical and demographic characteristics of the subgroups of patients based on their HoNOS trajectories.

HoNOS subgroups	Total number of patients	Psychotic disorder	Mood disorder	Substance- use disorder	Anxiety disorder	Personality disorder	Number of diagnoses	Gender	Age	Quality of life (baseline)	CANSAS (baseline)
	N	N (%)	N (%)	N (%)	N (%)	N (%)	Median number of diagnoses (IQR)	Male (%)	Years (SD)	Total score (SD)	Median number of unmet needs (IQR)
Stable at a low problem- severity level	709	512 (72%)	147 (21%)	126 (18%)	68 (10%)	79 (11%)	1 (1–2)	423 (60%)	42.9 (11.1)	37.8 (6.2)	2 (1–4)
Stable at a low medium problem-severity level	1208	775 (64%)	270 (22%)	273 (23%)	212 (18%)	198 (16%)	2 (1–2)	693 (57%)	43.3 (10.6)	33.4 (7.3)	4 (2–7)
Stable at a high medium problem-severity level	528	318 (60%)	129 (24%)	156 (30%)	115 (22%)	96 (18%)	2 (1–3)	331 (63%)	43 (10.7)	29.3 (7.6)	6 (3–9)
Stable at a high problem- severity level	116	66 (57%)	27 (23%)	43 (37%)	35 (30%)	23 (20%)	2 (1–3)	77 (66%)	44 (9.8)	26.3 (7.7)	9 (6–12)
Ameliorating subgroup	42	25 (60%)	13 (31%)	9 (21%)	9 (21%)	7 (17%)	2 (1–2)	25 (60%)	41.8 (10.6)	27.5 (5.4)	7 (4–11)
Deteriorating subgroup	57	33 (58%)	18 (32%)	26 (46%)	11 (19%)	14 (25%)	2 (1–3)	40 (70%)	46.3 (10.6)	33.2 (9.2)	4 (2–6)
Total	2660	1729 (65%)	604 (23%)	633 (24%)	450 (17%)	417 (16%)	2 (1–2)	1589 (60%)	43.2 (10.7)	33.5 (7.8)	4 (2–7)
Statistical test		chi2 = 27.053, df = 5, p < .001*	chi2 = 6.67, df = 5, p = .239*	chi2 = 51.131, df = 5, p < .001*	chi2 = 51.642, df = 5, p < .001*	chi2 = 18.946, df = 5, p = .002*	chi2 = 101.871, df = 5, p < .001	chi2 = 9.446, df = 5, p = .093*	F = 1.379, df = 5, p.229**	F = 93.005, df = 5; 161.543 p < .001***	chi2 = 381.857, df = 5, p < .001****

* Pearson's chi2 test

** One-way ANOVA

*** Welch's ANOVA

**** Kruskal Wallis test

At each assessment, HoNOS total scores were strongly correlated with total QoL scores (*r* ranging between .48 and .49, p < .001). Likewise, relationships between HoNOS and CANSAS total scores were moderate to strong (*r* ranging from .34 at the first assessment to .46 at the third assessment). Thus, the lowest quality of life and the greatest number of unmet needs were reported by patients who had the most psychosocial problems assessed using the HoNOS.

### Unmet needs for care at baseline

At baseline, the highest number of unmet needs was reported by patients in the subgroup with a stable high HoNOS trajectory (median 9; IQR 6–12), followed by those in the subgroup with an ameliorating HoNOS trajectory (median 7; IQR: 4–11). There was no significant difference between the stable high and ameliorating subgroups regarding the number of unmet needs at baseline. Therefore we had analysed differences in the individual needs in order to identify a pattern of needs that might discriminate between patients in the subgroup with a stable high HoNOS trajectory (severe and chronic problems) and those whose psychosocial problems improved over time. At the level of individual needs, we found that, at baseline, more patients in the subgroup with a stable high HoNOS trajectory reported unmet needs with regard to Physical Health problems (52%) than those in the subgroup with an ameliorating HoNOS trajectory (33%, Chi2(1) = 3.907, p = 0.048). We also found that more patients in the subgroup with a stable high HoNOS trajectory than in the subgroup with an ameliorating HoNOS trajectory reported unmet needs regarding Accommodation (42% vs. 23%, Chi2(1) = 4.401; p = 0.036); Food (42% vs. 18%, Chi2(1) = 7.182, p = 0.007); Transportation (22% vs. 7%, Chi2(1) = 3.826, p = 0.050); and Telephone (11% vs. 0%, Chi2(1) = 4.643, p = 0.031; Fisher Exact Test). There were no differences regarding other needs.

At baseline, the patients in the subgroup with a stable low HoNOS trajectory reported the lowest number of unmet needs (median 2; IQR: 1–4). They were followed by the patients in the subgroups with stable low medium and deteriorating HoNOS trajectories. As patients in both of the latter groups reported a median of four unmet needs, we compared the two groups with regard to individual needs. This showed that, at baseline, more patients in the subgroup with a deteriorating HoNOS trajectory had reported unmet needs involving problematic alcohol use than patients in the stable low medium HoNOS trajectory (12% vs. 5%, Chi2(1) = 4.337, p = 0.050, Fisher Exact test). We also found a trend that more patients in the subgroup with the stable low medium HoNOS trajectory reported problems with psychotic symptoms (20% vs. 10%, Chi2(1) = 2.764, p = 0.096), although not significantly. We found no other differences regarding unmet needs.

### Change in unmet needs over time (Table 3)

Overall, unmet needs decreased over the two-year treatment period from four unmet needs per patient to three (WSRT = -10,861; p<0.001). Except for patients in the subgroup with a deteriorating HoNOS trajectory, all trajectories reported a decrease in unmet needs. In the subgroup with a deteriorating HoNOS trajectory, unmet needs per patient increased from three needs to seven.

**Table 3:** Unmet needs of patients in six subgroup trajectories consisting of homogenous groups of SMI patients in contact with a FACT team over a two-year period.

HoNOS subgroups	Total number of patients	CANSAS (baseline)	CANSAS (assessment 3)	Wilcoxon Signed RankTest (Standardized Test statistic)	CANSAS Items**** showing significant change between assessment 1 and 3.*
	N**	Median number of unmet needs (IQR)	Median number of unmet needs (IQR)		
Stable at a low problem severity level	570	2 (1–4)	1 (0–3)	-5.015; p<0.001	1, 5, 7, 8, 9, 10, 14, 15, 19, 24
Stable at a low medium problem severity level	899	4 (2–7)	3 (1–6)	-8.700; p<0.001	2, 5, 7, 8, 9, 10, 14, 15, 18, 20, 21, 22, 23, 24, 27
Stable at a high medium problem-severity level	355	6 (3–9)	5 (2–8)	-4.457; p<0.001	2, 5, 8, 9, 10, 14, 18, 20, 21, 24, 26
Stable at a high problem- severity level	73	9 (6–12)	8 (5–11)	**-**2.359; p = 0.018	2, 22
Ameliorating subgroup	33	7 (4–11)	2.5 (1–5.75)	-2.988; p = 0.003	4, 7, 10, 14, 18, 25
Deteriorating subgroup	41	3 (2–6)	7 (3–12.25)	2.447; p = 0.014	3, 4, 7, 12, 13
Total	1971	4 (2–7)	3 (1–6)	-10.861; p<0.001	1, 2, 5, 7, 8, 9, 10, 12, 14, 15, 17, 18, 20, 21, 22, 23, 24, 27
Statistical test		Chi2 = 364.771, df = 5, p<0.001***	Chi2 = 314.971, df = 5, p<0.001***		

* McNemar test for related samples

** Number of patients, 689 patients had missing cansas assessments

*** Kruskal Wallis test

**** CANSAS items: 1 Accommodation; 2 Food; 3 Housekeeping; 4 Self-care; 5 Daytime activities; 6 Physical health; 7 Psychotic symptoms; 8 Information on condition and treatment; 9 Psychological distress; 10 Personal safety; 11 Safety to others; 12 Alcohol; 13 Drugs; 14 Companionship; 15 Intimate relationships; 16 Sexual Expression; 17 Child care; 18 Education; 19 Telephone; 20 Transport; 21 Money; 22 Benefits; 23 Paid work; 24 Side effects medication; 25 Recovery; 26 Legal problems; 27 Sleep

Patients in the subgroup with a deteriorating trajectory reported an increase in needs regarding Housekeeping, Self Care, Psychotic Symptoms, Alcohol, and Drugs. Patients in the ameliorating trajectory subgroup reported a decrease in needs regarding Self Care, Psychotic Symptoms, Personal Safety, Companionship, Education and Recovery.

The fewest unmet needs to decrease significantly were in the subgroup of patients with a stable high HoNOS trajectory. In this subgroup the expression of unmet needs for Food decreased from 36% of patients to 20%; and Social Benefits decreased from 16% of patients with unmet needs to 5%. Patients in the subgroup with a stable low HoNOS trajectory made progress regarding Accommodation, Daytime Activities, Psychotic Symptoms, Information on Condition and Treatment, Psychological Distress, Personal Safety, Companionship, Intimate Relationships, Telephone, and Side Effects of Medication.

Changes in needs in three subgroup–stable low, stable low medium and stable high medium–had more similarities than differences. So while the patients in question may have suffered from a different severity of symptom and social problems or had a different cluster of symptoms, they reported the same (changes) in needs for treatment.

## Discussion

On the basis of similarities in patients’ subgroup trajectories of psychosocial problems over a two-year period, we identified six homogenous patient groups in a large ROM dataset containing 2,660 patients with an SMI. As well as a subgroup of patients with a stable and low severity of problems (N = 709; 27%), we identified one subgroup of patients with a stable and low medium severity of problems (N = 1,208; 45%); one with a stable and high medium severity of problems (N = 528; 20%); one with a stable and high severity of problems (N = 116; 4%); one in whom problems had ameliorated (N = 42; 2%), and one in which they had deteriorated (N = 57; 2%).

The vast majority of patients (96%) can thus be clustered in one of the subgroups in which problems had remained stable, suggesting that the overall level of psychosocial problems had remained largely unchanged over two years of treatment. While this is in line with previous research [7; 40], the results also showed a significant decrease in the number of unmet needs within these relatively stable patient groups.

It is interesting that the subgroup of patients with a stable low, stable low medium and stable high medium trajectory had more similarities than differences with regard to decreases in the same individual needs. So, although patients in these trajectories differed–both at baseline and after two years of treatment–with regard to their levels of psychosocial problems and their numbers of unmet needs, this suggests that these patients tend to benefit in the same manner from contact with a FACT team. Within these stable trajectory subgroups, patients do thus achieve gains in terms of a reduction in unmet needs for care. However, a two-year period may not be enough to achieve significant improvement in terms of their psychosocial problems. On the other hand, in the case of some patients the stability of their problems may also reflect the chronic nature of their psychiatric symptoms. Or in some cases it may also due to the low expectations and ambitions of their clinicians [41–43].

In our study we were able to distinguish four distinct subgroups with stable trajectories in terms of severity of psychosocial problems, ranging from just above clinical cut-off to maximum severity of psychosocial problems. The combination of a large proportion of stable patients and distinct homogenous patient groups may indicate an interrelationship between the severity and type of psychopathology symptoms and social problems (HoNOS scores)–in other words, that these items influence each other and may form a self-sustaining cluster of symptoms and social or behavioural problems [44–45]. From a treatment perspective, this clustering of symptoms might indicate that a patient’s whole psychosocial functioning could be leveraged by targeting core problems.

As expected, we were able to distinguish a small proportion of patients who underwent a remarkable change over a two-year period: a subgroup who deteriorated over time, presenting a consistent increase in problems; and a subgroup whose condition ameliorated over time, with a consistent decline in psychosocial problems. We found that patients in the subgroup with the most psychosocial problems (as rated by their clinician) also reported the worst quality of life and the highest number of unmet treatment needs (patient perspective); and–mutatis mutandis–patients in the subgroup with the least psychosocial problems reported the best quality of life and the lowest number of unmet treatment needs [46].

### Differences between patient subgroups

Most patients with stable and low severity of psychosocial problems were diagnosed with just one DSM IV diagnosis, reflecting the low complexity of this subgroup, which may thus constitute a relatively low-maintenance group. More patients in this subgroup were diagnosed with a psychotic disorder than those in the other subgroups. This may mean that patients with this type of psychopathology tend to remain in contact with a FACT team, possibly because they may still need their medication or support from the FACT team (e.g. to prevent relapse). On the other hand, our clinical experience suggests that patients with a different cluster of symptoms–such as a patient with a personality disorder–were discharged more easily when the severity of their psychosocial problems were stable and low. Whether these patients are best treated in secondary care, primary care or GP care is a matter for debate. Decisions will depend partly on the availability and quality of care in a patient’s geographical region.

Interestingly, most patients within the stable high subgroup and the deteriorating subgroup were also diagnosed with a psychotic disorder, but were also more frequently diagnosed with a co-morbid substance-use-related disorder, anxiety-disorder and/or personality disorder. It thus appears that high levels of comorbidity and social problems have a poorer prognosis for treatment, presumably because comorbidity aggravates and complicates psychopathology it also worsens the course of the psychiatric disorder [47–50]. Examination of this group in greater detail–at the level of an individual’s unmet needs–shows that patients within the stable high subgroup reported the fewest decrease in unmet needs. Relative to patients in the ameliorating subgroup, these patients have more unmet needs regarding their somatic condition and their basic and practical needs (Accommodation, Food, Transport and Telephone). With regard to basic, psychiatric and/or rehabilitation needs, these patients benefited little or not at all from two years of contact with a FACT team. It may not therefore be beneficial for them to continue treatment in the same manner. If contact with a FACT team is not sufficient and is not helping the patient, the FACT team should discuss why the patient is not responding to treatment, and should consider two options: 1) stopping treatment, waiting for a more opportune moment and taking a watchful waiting attitude in consultation with the general practitioner; or otherwise 2) adopting a different therapeutic approach (e.g. more assertive, directive or supportive) and/or looking for dangerousness criteria that will trigger involuntary admission followed by treatment.

These choices about treatment are complex and consulting the outcomes on the ROM measures is a helpful step in this process as it provides valuable insights into a patient’s progress or stagnation, or otherwise into the complexity of his or her psychosocial problems. This can support the process of deciding on complex situations in therapy [51–52].

More specifically, a ROM assessment may also help indicate why a patient is not improving–if, for instance, his or her expression of several basic and practical unmet needs constitutes self-sustaining psychosocial problems [53]. Such understanding may help to formulate a treatment plan that is more comprehensive and more consistent with the patient’s needs [54]. Or, if the treatment has been targeted on these needs, outcome measures can support the discussion about the added value of treatment.

Our results also show that more unmet needs regarding problematic alcohol use were reported by patients who, before their relapse into psychosocial problems, had deteriorated during their contact with FACT. Our results also show that, at baseline, more of these patients had been diagnosed with a co-morbid substance use disorder than patients with a comparable level of psychosocial problems.

The differences in their diagnoses and unmet needs are strong indications that problems with substance use (such as alcohol) may be a risk factor for deterioration and are important symptoms that may aggravate other symptoms and social problems, increasing basic and psychiatric unmet needs over time. It is therefore important to detect any unmet needs for substances in a timely manner. Routine collection and discussion of outcome measures such as the HoNOS and CANSAS help to inform clinicians about a patient’s risk of deterioration, and also, provide opportunities for adjusting the treatment on time.

The differences in diagnoses between the subgroups also provides some evidence that the current FACT teams may be better equipped for providing treatment for patients with primarily psychotic disorders than for patients with personality disorders and substance-use disorders. Treatment for patients with personality disorders and substance use requires specialized treatment programs which may be difficult to fully implement within relatively small FACT teams. In our experience, professionals within FACT teams are good at making contact with these patients, providing pharmacotherapy, support and crisis contacts, but not all FACT teams are equipped to deliver specialized services (for personality disorders or a substance-use-related disorder) or to provide the appropriate levels of care. One solution may be to establish FACT teams specialized in the treatment of personality disorders and substance-use disorders.

## Limitations and strengths

Our study has two notable strengths. The first is that our use of a large sample enabled us to conduct statistical analyses that were dependent on large sample sizes. It also enabled us to detect smaller subgroups and small differences in clinical characteristics between subgroups. The second strength is that our study comprised a sample of SMI patients that was very similar to the patient population encountered by FACT teams in clinical practice.

Our study also had some limitations. Only 39% of the patients treated in a FACT team over the 2011–2016 period met our inclusion criteria. The other 61% were treated less than two years continuously, or had missed ROM-assessments. As a result, women and patients with mood and anxiety disorders were underrepresented in our analytical sample. We also found that included patients reported less severe psychosocial problems than those who had been excluded, although the clinical relevance of a one-point difference on the HoNOS total score is a matter of debate. Exclusion of these patients may have led to an underestimation of the prevalence of patients in the stable high subgroups. And possibly, we have missed the existence of subgroups of patients with (relatively) short treatment duration or low treatment adherence. We explicitly refrained from re-analysing our latent class model in the full ROM database (N = 6,897 patients, with a total of 20,925 assessments), since the data did not meet the assumptions necessary to reliably perform a MLR with missing data (i.e. excluded patients were not missing at random, and non-normality of the missing assessments) [55–56]. That being said, the validity of the six-patient trajectory subgroups we distinguished over a two-year period was not affected by the selection, and the social-demographic characteristics of the study sample closely resembled those of the Dutch SMI population at large [57–58].

A further limitation is that, despite our selection of a two-year treatment period, we were unable to distinguish long-term treatment effects, and to study whether new subgroups of patients emerge when account is taken of short-term and long-term treatment effects. And despite the size of our study sample, the number of patients with an ameliorating and deteriorating subgroups are small. Statistical inference regarding these small subgroups may have lacked power. Also our study has exploratory elements, for instance we examined which changes in needs were related to different subgroups of HoNOS trajectories. These exploratory analyses made multiple testing unavoidable, therefore we acknowledge the increased likelihood of false-positive results. So we recommend future studies to test these hypotheses and confirm the results from these analyses. Although the outcome measures (HoNOS, CANSAS we have used in our study are important to determine treatment success, future studies should consider to collect additional information on contact frequency, hospitalizations, court orders, guardianship, employment, income, assisted living or homelessness which may provide further insights in the context of these outcomes. Finally, due to the observational nature of our study, we are not able to infer causality.

## Conclusion

At the individual level, outcome measures such as the HoNOS and CANSAS have relevant evaluative and predictive value for patients with SMI. They can also be used to support treatment decisions. We found a strong indication that expressing a need for care concerning alcohol at baseline is a risk factor for deterioration.

At the group level, we found that higher levels of comorbidity (substance-use disorder, personality disorder or an anxiety disorder) suggest a poorer prognosis over time. A possible solution to this problem is to establish FACT teams specialized in the treatment of personality disorders and substance-use disorders. Finally, even though the overall level of psychosocial problems of SMI patients in contact with FACT teams remains relatively stable, these patients achieve gains in terms of the number of unmet needs.
